# Ebola Virus in Breast Milk in an Ebola Virus–Positive Mother with Twin Babies, Guinea, 2015

**DOI:** 10.3201/eid2204.151880

**Published:** 2016-04

**Authors:** Helena Nordenstedt, Elhadj Ibrahima Bah, Marc-Antoine de la Vega, Mamadou Barry, Magassouba N’Faly, Moumié Barry, Beatrice Crahay, Tom Decroo, Michel Van Herp, Brecht Ingelbeen

**Affiliations:** Karolinska Institutet, Stockholm, Sweden (H. Nordenstedt);; Médecins Sans Frontières Operational Centre Brussels, Conakry, Guinea (H. Nordenstedt, E.I. Bah, M.-A. de la Vega, Mamadou Barry, B. Crahay, B. Ingelbeen);; Donka National Hospital, Conakry (E.I. Bah, Moumié Barry);; University of Manitoba, Winnipeg, Manitoba, Canada (M.-A. de la Vega);; Université Gamal Abdel Nasser, Conakry (M. N’Faly);; Médecins Sans Frontières Operational Centre Brussels, Brussels, Belgium (T. Decroo, M. Van Herp);; Institute of Tropical Medicine, Antwerp, Belgium (B. Ingelbeen)

**Keywords:** Ebola virus disease, breast milk, lactating, Ebola virus, Guinea, virus, Guinea, viruses, twins, transmission, outbreak

**To the Editor:** Field clinicians working during the unprecedented Ebola virus disease (EVD) outbreak in West Africa, which began in December 2013, have been confronted with complex situations concerning mothers and breast-fed children in which one or both in a pair have tested positive for Ebola virus (EBOV) (*1*). More data, especially regarding virus shedding in breast milk, is critical to provide better care and guidance in future outbreaks. We report the case of a lactating EBOV-positive mother and her twin babies. The case is anonymously reported with the mother’s consent. The study met the Médecins Sans Frontières Ethics Review Board and approved criteria for studies of routinely collected data.

In Guinea in 2015, a woman and her 4-month-old twins (baby 1 and 2) were registered as contacts after the woman’s mother tested positive for EBOV (postmortem diagnosis by reverse transcription PCR [RT-PCR]). The woman and her babies, who were exclusively breast-fed, were followed daily by contact tracers. When baby 1 became febrile, the woman left her home to seek help from a traditional healer, bringing both twins with her. A few days later, baby 1 died and was buried without EBOV testing; according to the World Health Organization case definition, baby 1 was a probable EVD case-patient (*2*). 

Eleven days after baby 1 died, the woman became sick; 5 days later, she was admitted to an Ebola treatment center. At admission (day 0), she had headache, loss of appetite, abdominal pain, joint pain, dysphagia, conjunctival injection, and myalgia but was afebrile. On day 1, a blood sample from the woman was positive for EBOV by RT-PCR (Xpert Ebola Assay, GeneXpert Instrument Systems; Cepheid, Sunnyvale, CA, USA) with a cycle threshold (C_t_) of 32.5. Baby 2 tested negative for EBOV on day 1 and 72 hours later. Baby 2 was tested twice because he was considered at high risk for infection after being breast-fed for 6 days while his mother was symptomatic (i.e., until day 1 of her hospital admission).

On day 1, the woman was given convalescent-phase plasma from EBOV survivors; the treatment was given according to a compassionate-use protocol and was the standard process in this center at the time. On day 6, breast milk was sampled and tested positive for EBOV (C_t_ 21.6) (Table). The woman’s clinical course was favorable; she remained afebrile during hospitalization, but mild symptoms persisted until day 5. The first convalescent-phase test, done on day 14, showed C_t_ values of 40.5 and 27.5 for blood and breast milk, respectively. On day 21, a second breast milk sample tested positive (C_t_ 32.7). On day 24, the woman was given cabergoline (0.5 mg 2×/d for 2 days) to cease lactation, after which no more breast milk samples could be collected. On day 29 after admission, she tested negative for EBOV in blood and urine and was reunited with baby 2. Serologic testing for baby 2 was done on day 23 and showed no sign of previous subclinical infection (ELISA, IgM, and IgG negative). 

**Table:** Overview of results from all Ebola virus RT-PCRs performed during hospitalization of breast-feeding mother of twin babies, Guinea, 2015*

Day after admission	Blood, C_t_	Breast milk, C_t_	Urine, C_t_
1	32.5, glycoprotein	NT	NT
3	33.7, glycoprotein	NT	NT
6	NT	21.6, nucleoprotein	NT
14	40.5, nucleoprotein	27.5, nucleoprotein	NT
18	41.0, glycoprotein	NT	NT
21	40.3, nucleoprotein	32.7, nucleoprotein	NT
25	39.3, nucleoprotein	NT	NT
29	Negative, glycoprotein and nucleoprotein	NT	Negative, glycoprotein and nucleoprotein

*Testing performed by using the Xpert Ebola Assay (GeneXpert Instrument Systems, Cepheid, Sunnyvale, CA, USA). The lowest of the reported glycoprotein and nucleoprotein values are reported. C_t_ values <20 are highly positive, whereas C_t_ values >35 are weakly positive. C_t_, cycle threshold; NT, not tested.

Many questions in this case remain unanswered, but our findings show the potential infectivity of breast milk for at least 26 days after EVD symptom onset and demonstrate a case in which a baby was not infected by breast milk from his EBOV-positive mother. However, it should be noted that the woman’s breast milk was never tested while she was breast-feeding baby 2.

The literature on EBOV in breast milk of EBOV-positive patients is extremely scarce (*3*). In a previous study from the 2000 Sudan EBOV outbreak in Gulu, Uganda, breast milk from a convalescent-phase patient was sampled 15 days after symptom onset and tested positive for EBOV by RT-PCR and virus culture (*4*). Another study conducted in Guinea during the current outbreak, reported a mother–baby pair in which EVD developed in the baby 14 days after symptom onset in the mother, but breast milk from the mother sampled 17 days after symptom onset was negative by EBOV RT-PCR (*1*). 

It is unclear whether infectious virus or defective particles are being secreted in breast milk. C_t_ values were consistently lower in breast milk than in blood when tested concomitantly, but in this case, breast milk samples were not collected until day 6. Our findings suggest that breast milk is infected by EBOV at a later stage of the disease than blood but then follows the expected replication kinetics observed in venous blood.

Considering the high EVD death rate, until further evidence is found, we recommend that EBOV-positive women stop breast-feeding immediately and that breast-feeding not be resumed until 2 negative RT-PCR tests of the breast milk have been confirmed. This suggestion is in line with the World Health Organization recommendation for testing semen in male EVD survivors (*5*). The public health risk for EBOV to remain in breast milk for at least 26 days after EVD symptom onset and for breast milk to possibly be infectious after a patient has cleared the virus from the blood should also be acknowledged.
